# Effects of Methionine‐Supplemented Low‐Protein Diets on Production Efficiency, Heat Resilience, and Welfare of Broilers in Tropical Climates

**DOI:** 10.1002/vms3.70512

**Published:** 2025-07-28

**Authors:** Tchabltien Songuine, Lochina Feteke, Cocou Claude Kpomasse, Tchablémane Yarkoa, Tchilabalo Parobali, Simplice D. Karou, Wéré Pitala

**Affiliations:** ^1^ Regional Center of Excellence on Avian Sciences (CERSA), University of Lome Lome Togo; ^2^ Faculty of Health Sciences, University of Lome Lome Togo

**Keywords:** gait score, heat stress, H/L ratio, litter quality, methionine, welfare

## Abstract

**Background:**

The feeding strategy is one of the alternatives for countering the harmful effects of heat stress in broilers. This study was carried out to evaluate the effect of low‐protein diets supplemented with different levels of dietary methionine on the growth performance and welfare of Cobb500 broilers raised in a tropical climate.

**Materials and Methods:**

A total of 500 15‐day‐old Cobb500 broilers were randomly divided into five treatments with five replicates of 20 chickens each: chickens fed a standard methionine‐free diet (ME: 3100.97 kcal/kg) with high crude protein (P) content (18.25%) (T^+^) and low P content (15%) (T^−^) and chickens fed a low P diet (15%) supplemented with methionine at rates of 0.05% (M_1_), 0.08% (M_2_), and 0.1% (M_3_). The trial lasted for 28 days, during which water and feed were provided ad libitum. Growth performance and carcass composition were evaluated. Gait abnormalities and litter quality were assessed at 38 and 42 days of age, respectively. At 45 days of age, blood samples were collected, and the serum was used for biochemical and physiological parameter analysis.

**Results:**

Chickens in the M_3_ treatment showed higher growth performance (*p* < 0.05) than those in the other treatments. The thyroid hormone concentrations were higher (*p* < 0.05) in the M_3_ treatment, and litter quality is good in all birds fed a low‐protein diet supplemented with methionine.

**Conclusion:**

Methionine supplementation at the level of 0.1% to a 15% protein diet improved the growth performance and welfare of Cobb500 broilers under a tropical climate.

## Graphical abstract

Methionine supplementation increased feed intake, reduced abdominal fat and total cholesterol levels, and improved physiological parameters in broilers. Thus, methionine supplementation to a low‐protein feed improves production efficiency, heat resilience, and meat quality in broilers reared in hot, humid conditions.

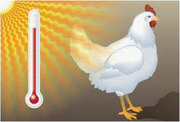

## 1 Introduction

Global warming is a serious and real issue that demands attention and action and should not be disregarded. (Wang et al. 2023; Chowdhury et al. 2021). According to Renaudeau et al. (2012), tropical conditions limit the growth performance of broiler chickens. In fact, the functional ability of temperature regulation is most effective when birds are kept within a thermoneutral zone, ranging from 21 to 28°C, which allows them to maintain stable internal organ temperatures (Apalowo et al. 2024). Studies have shown that environmental temperatures exceeding 25°C can induce heat stress in poultry (Shakeri et al. 2020). This is a major challenge in tropical climates and has a negative impact on poultry production, affecting production performance, liveability, and immune functions in birds (Kpomasse et al. 2021). Faced with this challenge, several performance‐enhancing strategies were then developed, including dietary strategies that consist of amino acid supplementation with a low‐protein feed (Attia et al. 2020a; Attia et al. 2020b). Methionine is commonly supplemented in broiler diets to meet the requirements for sulphur‐containing amino acids (SAA), essential for optimal growth performance (Zeitz et al. 2020). It plays a crucial role in the antioxidant system and serves as a precursor to cysteine (Cys) (Ingenbleek and Kimura, 2013), which in turn is a precursor or component of important antioxidants like taurine and glutathione (GSH) (Mari et al., 2009). Dietary supplementation of methionine may be particularly beneficial when broilers are exposed to heat stress, as it increases the availability of methionine metabolites that contribute to the body's antioxidant defence system (Zeitz et al., 2020). Also, methionine has been studied for its potential to mitigate the effects of heat stress (Santana et al., 2021). It is a vital amino acid for protein synthesis and has antioxidant properties, acting as a precursor to cysteine and glutathione in the transsulfuration pathway (Swennen et al. 2011). Methionine has also been reported to reduce air pollution during poultry production and improve broiler welfare. Findings of Attia et al. (2020a) indicated an improvement in carcass yield and a 21% reduction in nitrogen excretion by supplementing methionine from 0.08% to 0.1% in a low crude protein diet (15%). Additionally, Hossain et al. (2017) reported that broilers fed a high‐protein supplemented diet exhibited poorer growth performance, which was attributed to reduced nutrient digestibility. It is well known that crude protein contributes to increased metabolic heat production due to its higher heat increment compared with fats and carbohydrates (Wang et al. 2022). Therefore, reducing dietary protein levels not only conserves protein resources and reduces nitrogen excretion but also lowers breeding costs, diminishes pathogenic bacteria, and improves intestinal health (Wang et al., 2022). However, free amino acids, which do not require enzymatic digestion, do not contribute to the heat produced during digestion (Morales et al. 2020).

This experiment was designed to assess the effect of low‐protein diets supplemented with different levels of dietary methionine on the growth performance, thermotolerance, meat quality, biochemical parameters, and welfare of broiler chickens raised in tropical climates.

## 2 Materials and Methods

### 2.1 Study Site

The experiment was carried out at the experimental unit of the Regional Center of Excellence on Avian Sciences (CERSA) in Badja with precise geographic coordinates, coordinates: 6° 23' 0“ North, 0° 59' 0” East, 41 km from Lome, Togo, located in a hot and humid climate. The average temperatures recorded during the experiment varied from 26.87°C ± 0.28 to 30.83°C ± 0.95, with an average of 28.85°C ± 0.62, while the relative humidity varied from 65.23% ± 3.19 to 78.01% ± 1.31, with an average of 71.62% ± 1.75.

### 2.2 Animals and Dietary Treatments

A total of 500 15‐day‐old Cobb 500 broiler chicks were randomly divided into five treatments of five replicates of 20 chickens each using a completely randomised design (CRD). The chickens were fed a standard methionine‐free diet (ME: 3100.97 kcal/kg) with high crude protein content (18.25%) (T^+^) and low crude protein content (15%) (T^‐^). The chickens were fed a low crude protein diet (15%) supplemented with methionine at rates of 0.05% (M_1_), 0.08% (M_2_), and 0.1% (M_3_). The broiler chickens were raised at a density of 10 birds per m^2^. They were reared on a deep litter (wood shavings) floor in an open‐sided poultry house. The trial lasted for 28 days, during which water and feed were provided ad libitum. All the diets were formulated according to the Cobb500 broiler's nutritional needs (Table 1). The lighting program consisted of a period of 23 h of light and 1 h of darkness (Songuine et al. 2024, Yarkoa et al. 2024). In this trial, four thermo‐hygrometers were used to measure daily temperature and relative humidity in the poultry house.

**TABLE 1:** Composition of the ingredients of the experimental diets.

Ingredients (%)	T^+^	T^‐^	M^1^	M^2^	M^3^
Maize	53.6	60	60	60	60
Wheat	14.6	14.2	14.2	14.2	14.2
Soybean	19	13	13	13	13
Oyster shell	1.5	1.5	1.5	1.5	1.5
Salt	0.3	0.3	0.3	0.3	0.3
Broiler concentrate (5%)^*^	5	5	5	5	5
Dried brewers grains	6	6	6	6	6
Methionine^1^	0.0	0.0	0.05	0.08	0.1
Chemical composition of the diet					
Metabolizable energy (MJ/kg) ^2^	12.84	12.84	12.84	12.84	12.84
Crude protein (g/kg)^3^	182.50	150.04	150.04	150.04	150.04

Broiler chicken fed a high‐protein methionine‐free diet (T^+^), a low‐protein methionine‐free diet (T^‐^), and a low‐protein diet supplemented with methionine at rates of 0.05% (M_1_), 0.08% (M_2_), and 0.1% (M_3_).

* Composition: Soybean meal, rapeseed meal, sunflower seed meal, corn gluten feed, vinasse, soybean oil, palm fatty acids, and sodium chloride. Vit. A, 12,000 IU; Vit. E, dl‐α‐tocopheryl acetate 20 mg; menadione 2.3 mg; Vit. D3, 2200 ICU; riboflavin 5.5mg; calcium pantothenate 12mg; nicotinic acid 50 mg; choline 250 mg; Vit. B12 10 ug; Vit. B6 3 mg; thiamine 3 mg; folic acid 1 mg; d‐biotin 0.05 mg. Trace minerals, mg/kg of diet: Mn 80, Zn 60, Fe 35, Cu 8, Selenium 0.1mg.

^1^ Methionine was used as a supplement.

^2^ Metabolizable energy was calculated.

^3^ Crude protein was analysed.

### 2.3 Analysis of Diets Composition

A sample of the diets used was subjected to bromatological analysis. The protein content of the diets was assessed by the Kjeldahl method in accordance with NF EN ISO 20483 (Joubert et al. 2018). These protein contents are presented in Table 1.

### 2.4 Growth Performance and Carcass Composition Evaluation

Growth performance indicators such as feed intake and body weight were measured during the experiment to determine the weight gain and feed conversion ratio (FCR).

After being fasted for 6 to 8 h before slaughter, six broiler chickens were randomly selected from each treatment at 45 days of age and were humanely sacrificed and eviscerated to take carcass data, including abdominal fat, carcass yield, breast yield, thigh yield, emptied gizzard, liver, heart, kidney, and intestinal weight and length. The individual weights were expressed as a relative weight of the body weight, according to Kim et al. (2016), as follows:

$$\mathrm{CY}\;\left(\%\right)=\frac{CP}{BW}\times 100,$$

where: CY (%) is carcass yield, CP is chicken carcass part (g) and BW is the body weight of the chicken before slaughter (g).

### 2.5 Biochemical Profiles and Physiological Responses to Heat Stress

At 45 days of age, approximately 3 mL of blood was collected from the wing veins of 15 animals per treatment before slaughter, discharged into a dry tube without anticoagulant, and immediately centrifuged at 3000 rpm for 15 min to obtain serum. Then, the serum obtained was stored in a freezer at ‐20°C (Kpomasse et al. 2020) and further used for biochemical parameter analysis such as total protein (Busher 1990), total cholesterol (Borner and Klose 1977), uric acid (Barr 1990), glucose (Heinz and Beushausen 1981), and triglycerides (Wahlefeld 1974), as well as physiological parameters such as hormone T3 (triiodothyronine), T4 (thyroxine) (Dunlap 1990), and H/L (heterophil/lymphocyte ratio) (Lentfer et al. 2015).

### 2.6 Meat Quality

#### 2.6.1 Meat PHu

At 45 days of age, the chicken's meat quality was assessed. Breasts carefully collected from chickens per treatment were kept at 4°C for 24 h. At the end of this period, the ultimate pH (pHu) of the muscle was measured by inserting a glass electrode directly into the thickest part of the *pectoralis major* using an OARTON pH 700 pH meter accurate to 0.01 (Beauclercq et al. 2022).

#### 2.6.2 Meat Exudation

Exudate (loss of fluid from the muscle without the application of mechanical force) was measured by weighing meat samples (Pi for initial weight) in plastic bags, then hung and after 24 h sample weights were recorded (Pf for final weight). Exudate was calculated using the following formula: %Exudate = [(initial weight (Pi)—final weight (Pf)) / initial weight (Pi)] × 100 (De Carvalho et al. 2018; Wilhelm et al. 2010).

#### 2.6.3 Cooking Loss

Cooking Loss (CL) was measured by weighing meat samples (Pi for initial weight) in plastic bags, then immersed in a 75°C water bath until the internal temperature (30 min) reached 75°C. After cooling, sample weights were recorded (Pf for final weight). Loss on cooking was calculated using the formula of Filho et al. (2017): %PC = [(weight before cooking (Pi)—weight after cooking (Pf)) / weight before cooking (Pi)] × 100.

### 2.7 Welfare Assessment

#### 2.7.1 Gait Score

Gait scores for 38‐day‐old birds were assessed using the Garner et al. (2002) system: 0 for normal, 1 for slight, unidentifiable abnormalities, 2 for identifiable abnormalities with limited impact, 3 for obvious abnormalities affecting movement, and 4 for severe abnormalities. A score of 5 was excluded. In the study, scores 2 and 3 represented moderate lameness, while 4 and 5 indicated severe lameness (Buijs and Tuyttens, 2016).

The prevalence of gait disorders was then determined using the following formula:

$$\begin{array}{ccc} & & \mathrm{Prevalence}\;\;\left(\%\right)\\ & & \;=\frac{\left(\mathrm{Total}\;\mathrm{no}.\;\mathrm{of}\;\mathrm{chickens}\;\mathrm{with}\;\mathrm{scores}\;3\;\mathrm{and}\;4\right)}{No.\;of\;live\;chickens\;at\;time\;of\;assessment}\;\;\times 100\% \end{array}$$

#### 2.7.2 Litter Quality Assessment

The assessments were carried out on birds aged 42 days using the Welfare Quality (2009) procedure and according to Tuyttens et al. (2015). The litter score, litter depth, and droppings weight were all calculated. At 15 days old, each enclosure received wood shavings to cover the entire floor, replaced every four weeks. Dropping amounts were determined by weighing the litter before and after evaluation. Enclosures were divided into four zones for inspection, with average values representing litter depth and texture. Texture was assessed on a 5‐point scale: (1) dry and friable, (2) slightly wet, (3) crusty in areas, (4) surface crusty but diggable, and (5) completely caked or wet (Bouassi et al. 2016).

### 2.8 Statistical Analysis

Data were analysed using the general linear model (GLM) procedure of GraphPad Prism 8 software v.8.02 (GraphPad Software, San Diego, CA, USA). The growth performance parameters, such as feed intake, body weight, weight gain, feed conversion ratio, slaughtering performance (carcass characteristics evaluation, abdominal fat weight, digestive organ weights, small intestine length and weight), meat quality, biochemical parameters, and litter depth and droppings weight, were subjected to one‐way analysis of variance (ANOVA). When significant, further analyses were performed using Tukeyʼs test (Benjamini and Braun 2002). Mortality was analysed with a χ2 test. Since abnormal scores and litter quality are ordinal variables, the Kruskall–Wallis test was used followed by the Mann–Whitney *U* test. All tested parameters were considered as significantly different at 5%. Data are presented as mean ± standard error of the mean.

## 3 Results

### 3.1 Effects of Methionine Supplementation in a Low‐protein Diet on Broiler Chicken Performance and Mortality

As indicated in Table 2, daily feed intake was higher in T^+^ and M_3_ birds compared to those groups of T^‐^, M_1_, and M_2_ birds. The body weight and weight gain of chickens fed a diet supplemented with methionine M_3_ were significantly higher (*p* = 0.0089) than T^+^, T^‐^, M_1,_ and M_2_. The feed conversion ratio (FCR) of the birds in M_2_ and M_3_ was lower (*p* = 0.0154) than in the M_1,_ T^+^, and T^−^ groups. Also, mortality was higher in T^+^ and T^‐^ birds (*p* = 0.019) than in those of M_1_, M_2,_ and M_3_.

**TABLE 2:** Effects of methionine supplementation to low protein diet on growth performances and mortality.

	Treatments					
Parameters	T^+^	T^‐^	M_1_	M_2_	M_3_	*p*‐value
Daily feed intake (g)	80, 51 ± 2,59^a^	70, 01 ± 1, 72 ^c^	70,65 ± 2,58^c^	74,48 ± 2,73^b^	81,44 ± 3,41 ^a^	0,0089
Body weight (g)	1715 ± 47,66^b^	1530± 55, 49^c^	1538± 52,14^c^	1729 ± 29,36^b^	1840± 24,13^a^	0,0009
Weight gain (g)	43,76 ± 0,90^b^	36, 23 ± 1, 38^c^	34,47 ± 0,97^c^	40,52 ± 0,48^b^	48,9 ± 4,13^a^	0,0058
Feed conversion ratio	1,82 ± 0,02^b^	1, 903 ± 0,05^a^	1,947 ± 0,01^a^	1,8 ± 0,01^b^	1,7 ± 0,03^b^	0,0154
Mortality (%)	2,167 ± 0,44 ^a^	1 ± 0,577 ^b^	0,04 ± 0,02 ^c^	0,05 ± 0,01 ^c^	0,01 ± 0,01 ^c^	0,0019

Abbreviations: *p*‐value: probability; ± SEM, standard error of means.

Broiler chicken fed a high‐protein methionine‐free diet (T^+^), a low‐protein methionine ‐free diet (T^‐^), and a low‐protein diet supplemented with methionine at rates of 0.05% (M_1_), 0.08% (M_2_) and 0.1% (M_3_).^a,b^: The means of different superscripts in the same row vary significantly (*p* < 0.05).

### 3.2 Effects of Methionine Supplementation in a Low‐protein Diet on Meat Yield and Abdominal Fat in Broiler Chickens

Table 3 presents the effect of methionine supplementation in a low‐protein diet on meat yield and abdominal fat. No significant difference was noticed in thigh and breast weights (*p* > 0.05) between the control and the other treatment groups. However, the carcass yield of the birds in the M_3_ treatment was higher (*p* = 0,0312) compared to those of the other treatment groups. Additionally, abdominal fat was significantly reduced (*p* = 0,0494) in birds of M_2_, M_3,_ and T^‐^ compared to that of the control group T^+^ and M_1_ group (Table 3).

**TABLE 3:** Effects of methionine supplementation to low protein diet on abdominal fat and meat yield.

	Treatments					
Parameters	T^+^	T^‐^	M_1_	M_2_	M_3_	*p*‐value
Carcass yield (%)	64,17 ± 0,08^b^	62,22 ± 1,22^b^	61,64 ± 1,83^b^	64,33 ± 1,57^b^	68,45 ± 1,78^a^	0,0312
Breast yield (%)	17,39 ± 0,50	17,84 ± 1,01	18,17 ± 0,44	18,09 ± 1,12	17,74 ± 1,06	NS
Thigh yield (%)	8,84 ± 0,01	9,29 ± 0,39	8,81 ± 0,42	8,94 ± 0,16	8,88 ± 0,19	NS
Abdominal fat (%)	1,73 ±0,29^a^	1,22 ± 0,30^b^	1,42 ± 0,06^ab^	1,37 ± 0,08^b^	0,94 ± 0,07^c^	0,0494

Abbreviations: NS, no significative; *p*‐value: probability; ± SEM, standard error of means.

Broiler chicken fed a high‐protein methionine‐free diet (T^+^), a low‐protein methionine ‐free diet (T^‐^), and a low‐protein diet supplemented with methionine at rates of 0.05% (M_1_), 0.08% (M_2_) and 0.1% (M_3_).^a,b^: The means of different superscripts in the same row vary significantly (*p* < 0.05).

### 3.3 Effects of Methionine Supplementation in a Low‐protein Diet on Internal Organ Weights

No significant difference was noticed in the kidney, bile, gizzard, heart, lung, or pancreas weights (*p* > 0.05) among all the treatment groups. Nevertheless, the heart weight of the birds in M_3_ was similar to that of T^+^ but significantly higher (*p* = 0, 0228) than that of T^‐^, M_1_, and M_2_. The liver and small intestine weights were also higher in M_3_ than those groups (Table 4).

**TABLE 4:** Effects of methionine supplementation to low protein diet on digestive organ weights and small intestine weight and length.

	Treatments					
Parameters	T^+^	T^‐^	M_1_	M_2_	M_3_	*p*‐value
Heart (%)	0,55 ± 0,12 ^a^	0,47 ± 0,09 ^b^	0,41 ± 0,08 ^c^	0,48 ± 0,01 ^b^	0,56 ± 0,02 ^a^	0,0228
Liver (%)	2,07 ± 0,07 ^b^	1,92 ± 0,14 ^c^	2,19 ± 0,15 ^b^	2,04 ± 0,25 ^b^	2,50 ± 0,19^a^	0.0354
Bile (%)	0,11 ± 0,03	0,11 ± 0,02	0,11 ± 0,01	0,08 ± 0,01	0,11 ± 0,02	NS
Kidney (%)	0,49 ± 0,05	0,41 ± 0,05	0,43 ± 0,02	0,46 ± 0,07	0,55 ± 0,04	NS
Pancreas (%)	0,32 ± 0,01	0,29 ± 0,03	0,38 ± 0,04	0,28 ± 0,03	0,36 ± 0,04	NS
Gizzard (%)	3,42 ± 0,17	4,22 ± 0,28	3,64 ± 0,15	3,40 ± 0,16	3,61 ± 0,19	NS
Lung (%)	0,55 ± 0,02	0,60 ± 0,03	0,52 ± 0,06	0,53 ± 0,08	0,58 ± 0,08	NS
Intestine weight (%)	4,45 ± 0,23^c^	5,12 ± 0,15^b^	5,35 ± 0,72^b^	5,72 ± 1,05^b^	6,73 ± 0,45^a^	0,0089
Intestine length (cm)	159,8 ± 1,28^b^	156,4 ± 4,00^b^	160 ± 7,90^b^	162,2 ± 5,23^b^	192,3 ± 4,4^a^	0,0043

Abbreviations: NS, no significative; *p*‐value: probability; ± SEM, standard error of means.

Broiler chicken fed a high‐protein methionine‐free diet (T^+^), a low‐protein methionine ‐free diet (T^‐^), and a low‐protein diet supplemented with methionine at rates of 0.05% (M_1_), 0.08% (M_2_) and 0.1% (M_3_).^a,b^: The means of different superscripts in the same row vary significantly (*p* < 0.05).

### 3.4 Effects of Methionine Supplementation in a Low‐protein Diet on Gait Score and Litter Quality

Table 5 shows the effects of methionine supplementation in a low‐protein diet on gait score and litter quality. The feeding strategy had a significant effect (*p* = 0.0107) on the abnormal gait. Litter quality was higher in broiler chickens fed a low‐protein diet supplemented with methionine but higher in T^+^ and T^‐^ chickens (*p* = 0.007). Additionally, dropping weight and litter depth of birds in T^+^ and T^‐^ were higher (*p* = 0,0133, *p* = 0,0046) compared to those in M_1_, M_2,_ and M_3_ birds.

**TABLE 5:** Effects of methionine supplementation to low protein diet on gait score and litter quality.

	Treatments					
Parameters	T^+^	T^‐^	M_1_	M_2_	M_3_	*p*‐value
Abnormal gait	2,16 ± 0,44^a^	2,43 ± 0,34^a^	1,16 ± 0,16^b^	1,16 ± 0,60^b^	1,07 ± 0,34^b^	0.0107
Prevalence (%)	5,28 ± 2,88	5,28 ± 2,88	3.36±0.833	5,28 ± 2,88	2,45 ± 0.833	NS
Litter score	2,33 ± 0,19^a^	1,83 ± 0,19^b^	1,01 ± 0,25^c^	1,08 ± 0,10^c^	0,66 ± 0,15^d^	0,0007
Litter depth (cm)	2,16 ± 0,16^a^	2,10 ± 0,28^a^	2,16 ± 0,44^a^	2,03 ± 0,28^a^	1,83 ± 0,44^b^	0,0046
Droppings weight (g/bird)	2,22 ± 0,09^a^	2,06 ± 0,19^a^	1,80 ± 0,07^b^	1,55 ± 0,08^c^	1,43 ± 0,17^c^	0,0133

Abbreviations: ± NS, no significative; *p*‐value: probability; ± SEM, standard error of means.

Broiler chicken fed a high‐protein methionine‐free diet (T^+^), a low‐protein methionine ‐free diet (T^‐^), and a low‐protein diet supplemented with methionine at rates of 0.05% (M_1_), 0.08% (M_2_) and 0.1% (M_3_).^a,b^: The means of different superscripts in the same row vary significantly (*p* < 0.05).

### 3.5 Effects of Methionine Supplementation in a Low‐protein Diet on Biochemical Parameters

As stated in Table 6, no difference was observed in glucose and triglycerides contents among all treatment groups (*p* > 0.05). Serum protein content was reduced (*p* = 0, 0062) with birds of T^‐^ and M_1_ than those in the treatment groups. Similarly, serum uric acid content was reduced (*p* = 0, 0055) with birds of M_2_ and M_3_. Groups T^+^ and T^‐^ had higher (*p* = 0, 0023) cholesterol content than the other three groups which were similar.

**TABLE 6:** Effects of methionine supplementation to low protein diet on biochemical parameters.

	Treatments					
Parameters	T^+^	T^‐^	M_1_	M_2_	M_3_	*p*‐value
Total Protein (g/l)	41,02 ± 0,58^a^	35,04 ± 1,15^b^	37,81 ± 0,73^b^	39,11 ± 0,98^a^	40,38 ± 1,22^a^	0,0062
Cholesterol (g/l)	1,752 ± 0,01^a^	1,46 ± 0,05^ab^	1,39 ± 0,03^b^	1,34 ± 0,03^b^	1,36 ± 0,05^b^	0,0023
Uric acid (mg/l)	50,47 ± 1,88^a^	48,69 ± 9,47^b^	46,46 ± 6,28^b^	38,31 ± 6,54^c^	35,88 ± 3,71^c^	0,0055
Glucose (g/l)	2,25 ± 0,04	2,06 ± 0,17	2,02 ± 0,08	1,94 ± 0,07	2,02 ± 0,08	NS
Triglycerides (g/l)	0,63 ± 0,03	0,54 ± 0,07	0,62 ± 0,19	0,60 ± 0,18	0,45 ± 0,14	NS

Abbreviations: NS, no significative; *p*‐value: probability; ± SEM, standard error of means.

Broiler chicken fed a high‐protein methionine‐free diet (T^+^), a low‐protein methionine ‐free diet (T^‐^), and a low‐protein diet supplemented with methionine at rates of 0.05% (M_1_), 0.08% (M_2_) and 0.1% (M_3_).^a,b^: The means of different superscripts in the same row vary significantly (*p* < 0.05).

### 3.6 Effects of Methionine Supplementation in a Low‐protein Diet on Immune Organ Weights, Thyroid Hormone Contents and Heterophil/Lymphocyte Ratio (H/L ratio)

Table 7 shows the effects of methionine supplementation in a low‐protein diet on the thymus and spleen weights and the physiological responses of broiler chickens. There was no difference (*p* > 0.05) in the bird's spleen weights. However, thymus weight was similar in M_1_, M_2,_ and M_3_ chickens but higher than (*p* = 0.0356) that of T^+^ and T^‐^ group birds. The blood T3 (triiodothyronine) and T4 (thyroxine) content was higher (*p* = 0, 0104, *p* = 0, 0108) in M_3_, M_2,_ and T^+^ birds than those of T^‐^ and M_1_. Finally, the heterophil/lymphocyte (H/L) ratio of the birds of the T^‐^ group was higher (*p* = 0.0302) than that of the other birds.

**TABLE 7:** Effects of methionine supplementation to low protein diet on physiological parameters.

	Treatments					
Parameters	T^+^	^T‐^	M_1_	M_2_	M_3_	*p*‐value
T3 (pmol/l)	7,182 ± 0,38^b^	6,423 ± 0,34^c^	6,21 ± 0,631^c^	7,14 ± 0,403^b^	8,382 ± 0,33^a^	0,0104
T4 (pmol/l)	9,51 ± 0,34^b^	8,932 ± 0,51^c^	8,63 ± 0,39^c^	9,13 ± 0,37^b^	10,86 ± 0,60^a^	0,0108
H/L	1,64 ± 0,02^b^	2,13 ± 0,278^a^	1,72 ± 0,28^b^	1,13 ± 0,151^c^	1,12 ± 0,386^c^	0.0302
Thymus (%)	0.24 ± 0.0123^b^	0.24 ± 0.0123^b^	0.29 ± 0.0123^a^	0.32 ± 0.015^a^	0.31 ± 0.015^a^	0.0356
Spleen (%)	0,16 ± 0,05	0,11 ± 0,03	0,11 ± 0,01	0,09 ± 0,01	0,1 ± 0,01	NS

Abbreviations: H/L, heterophil/ lymphocyte ratio; NS, no significative; *p*‐value: probability; ± SEM, standard error of means; T3: Triiodothyronine; T4: Thyroxine.

Broiler chicken fed a high‐protein methionine‐free diet (T^+^), a low‐protein methionine ‐free diet (T^‐^), and a low‐protein diet supplemented with methionine at rates of 0.05% (M_1_), 0.08% (M_2_) and 0.1% (M_3_).^a,b^: The means of different superscripts in the same row vary significantly (*p* < 0.05).

### 3.7 Effects of Methionine Supplementation in a Low‐protein Diet on Meat Quality Parameters

Table 8 shows the effect of methionine supplementation on poultry meat quality. The results showed that there were no differences between the ultimate pH (pHu) values of chicken meat and water loss by exudation between the different treatments. However, water loss through meat cooking was higher (*p* = 0, 0128) in chickens from M_2_ and M_3_ treatments compared with chickens from the other treatments.

**TABLE 8:** Effects of methionine supplementation to low protein diet on meat quality parameters.

	Treatments					
Parameters	T^+^	T^‐^	M_1_	M_2_	M_3_	*p*‐value
PHu	5, 56 ± 0,035	5, 65 ± 0,017	5,45± 0,01	5,53± 0,068	5,55 ± 0,023	NS
Meat exudation (%)	1, 5 ± 0,288	1, 167 ± 0,44	1,167± 0,44	1,5 ± 0,5	1,833 ± 0,726	NS
Cooking loss (%)	7, 467 ± 0,35 ^c^	7, 267 ± 1,79 ^c^	9,5 ± 0,68 ^b^	10,43 ± 2,43 ^a^	10,67 ± 0,29 ^a^	0,0128

Abbreviations: NS, no significative; *p*‐value: probability; PHu, ultimate pH; ± SEM, standard error of means.

Broiler chicken fed a high‐protein methionine‐free diet (T^+^), a low‐protein methionine ‐free diet (T^‐^), and a low‐protein diet supplemented with methionine at rates of 0.05% (M_1_), 0.08% (M_2_) and 0.1% (M_3_).^a,b^: The means of different superscripts in the same row vary significantly (*p* < 0.05).

## 4 Discussion

Thermal challenge due to global warming is one of the most important environmental concerns in broiler production that negatively affects their optimum productivity, requiring mitigation strategies such as feeding strategies (Kpomasse et al. 2021; Olgun et al. 2021). This study was carried out to evaluate the effect of methionine supplementation in a low‐protein diet on the production performance and welfare of broiler chickens reared in a hot and humid climate.

The results of the present study indicate that broilers fed low‐protein diets supplemented with 0.1% methionine recorded higher feed intake and consequently gained more weight than birds in the other treatments. Chickens weight is positively affected by the availability and balance of amino acids in the feed they consume (Baxter et al. 2020). According to Lisnahan et al. (2017), chicken growth could be improved by adjusting the levels of essential amino acids in the ration. So the improved performance of chickens in treatment M3 would probably be linked to the high methionine levels used. These results are similar to those of Attia et al. (2020a, b), who found that supplementing methionine from 0.08% to 0.1% in a low‐protein feed improved the growth performance of chickens in tropical climates. The improvement in feed efficiency recorded for the 0.1% methionine supplementation level in a low‐protein feed suggests that adequate amino acid supplementation is required to improve bird performance in the tropics (Awad et al., 2014; Attia et al., 2020).

Mortality was higher in T^+^ and T^‐^ birds than in the other treatment groups, suggesting physiological challenges like hyperthermia, causing cellular damage and organ failure, and dehydration, leading to electrolyte imbalances and organ failure (Kpomasse et al. 2021). Amino‐acid supplementation is often suggested to support broiler chickens during periods of heat stress due to its role in maintaining essential physiological functions, supporting growth, and enhancing immune response (Awad et al. 2014; Attia et al., 2020).

Thigh, breast, kidney, bile, gizzard, lung, and pancreas weights were not different in any of the treatment groups in this study. However, carcass weight was improved in M_3_ broiler chickens. This could be linked to the improved feed efficiency that led to a reduction in abdominal fat in these chickens. This might have improved the carcass weight of the chickens. According to Temim et al. (1999), high protein in broiler feed is transformed into fat deposits under tropical conditions. High levels of abdominal fat in broilers could be a major problem for meat quality, as shown by Jennen (2004). Also, in broiler chickens from treatment M_3_, the small intestine length was significantly increased, indicating morphological and histological changes (Buwjoom et al. 2010). The efficiency of dietary protein utilisation in poultry is influenced by gastrointestinal tract features, highlighting the importance of intestinal health for nutrient absorption and utilisation (Wang and Peng 2008).

In the present study, methionine supplementation influenced the gait score but not the prevalence of leg ache in chickens. Similar trends were observed by Ale Saheb Ale Saheb Fosoul et al. (2016). Metabolic diseases such as ascites and other disorders affect chicken welfare and are a loss in broiler production (De Smit et al. 2005). Chickens fed a low‐protein diet supplemented with methionine significantly reduced the score, litter depth, and droppings weight of thermally challenged chickens, slowing down litter degradation. High‐protein diets increase nitrogen excretion (Aletor et al. 2000), which has a negative impact on the environment. The improvement in litter quality in the treatments M_1_, M_2_, and M_3_ is thought to be due to the good protein synthesis in the feed, which reduced the rate of nitrogen excretion in the droppings. Ammonia excretion in droppings degrades litter quality. This confirms the work of Beski et al. (2015) who stated that the addition of synthetic amino acids to the poultry diet reduces nitrogen excretion in droppings in chickens. These findings might have contributed to the welfare of the broiler chickens, which is an important factor affecting meat quality (Marchewka et al. 2023). In fact, meat quality is a major concern for both poultry farmers and consumers. According to Mir et al. (2017), quantifiable meat properties such as water holding capacity, shear strength, drainability, cooking loss, pH, shelf life, and others are indispensable and provide added value to meat products. The results showed that there were no differences between the ultimate pH values of chicken meat and exudative water loss between the different treatments. However, water loss through meat cooking was higher in chickens from treatments M2 and M3 compared with chickens from the other treatments. Supplementing the feed with methionine can improve the water‐holding capacity of the meat. According to Apple and Yancey (2013), water holding capacity (WHC) is the inherent ability of meat to retain water when a force is applied to it (i.e., cutting, pressing, grinding, or packaging) or during meat processing (i.e., brining and cooking). Water retention capacity is used to assess meat quality. It expresses the strength with which water is bound to muscle proteins, or the relative quantity of water that is strongly bound to these proteins, and thus makes it possible to predict the meat's subsequent behaviour.

With regard to the present findings, serum glucose and triglyceride concentrations were not influenced by methionine supplementation. However, it did affect serum protein, uric acid, and cholesterol concentrations. Chickens fed a low‐protein feed supplemented with different levels of methionine had lower cholesterol concentrations than those of the control group. Cholesterol is a major lipid, the precursor of all steroid hormones and bile salts. Lipids are synthesised in the liver and stored in adipose tissue or abdominal tissue in the form of triglycerides (Hermier 1997). These fats contribute to the animal's fat content, which may result in poor meat quality in chickens.

The physiological response of chickens to heat stress is reflected in variations in various heat stress indicators. T3 and T4 hormone concentrations and thymus weight, as shown in this study, were higher in the M3 treatment groups, while the H/L ratio was lower compared to the other treatment groups. This suggests that methionine supplementation to a low‐protein feed had a positive effect on heat stress in chickens. The thymus is a lymphoid organ involved in non‐specific (non‐adaptive) and specific (adaptive) immune responses in birds (Reese et al. 2006). Heat stress led to a decrease in thyroid hormones (Gonzalez‐Rivas et al. 2019). Antioxidant enzyme activity is regulated by thyroid hormones (Kpomasse et al. 2023). This implies that improved concentrations of T3 and T4 hormones could be linked to better antioxidant status in broilers. The effect of feeding strategy on the heterophil/lymphocyte ratio could be explained by the fact that the heterophil/lymphocyte ratio is higher in heat‐stressed chickens (Mashaly et al. 2004; Bartlett and Smith 2003).

Methionine supplementation decreased mortality rates and enhanced carcass quality while increasing feed intake, weight gain, and feed efficiency. Furthermore, it had a favourable impact on litter quality, gait scores, and thymus weight and led to increased concentrations of T3 and T4 hormones. These results imply that methionine supplementation can improve metabolic balance, immunological response, and general well‐being, so successfully mitigating the negative effects of heat stress in broiler chicks.

## 5 Conclusion

Supplementing a low‐protein diet with 0.1% methionine significantly increased the Cobb500 broilers' body weight, weight gain, and feed conversion ratio. Additionally, this feeding strategy enhanced the quality of the litter, demonstrating its potential to maximise broiler performance and welfare in hot and humid climates. These results highlight the importance of amino acid supplementation techniques in reducing the negative consequences of dietary restrictions, especially in harsh environmental settings, and promoting effective and sustainable broiler chicken production.

## Author Contributions

**Tchabltien Songuine**: Conceptualisation, Methodology, Analysis, Data Curation, Writing (Original Draft). **Lochina Feteke**: Conceptualisation, Data Curation, Writing (Original Draft), Writing (Review and Editing), Supervision. **Cocou Claude Kpomasse**: Conceptualisation, Methodology, Analysis, Data Curation, Writing (Original Draft), Writing (Review and Editing). **Tchablémane Yarkoa**: Conceptualisation, Data Curation, Writing (Original Draft). **Tchilabalo Parobali**: Conceptualisation, Data Curation. **Simplice D. Karou**: Writing (Review and Editing) Supervision. **Wéré Pitala**: Writing (Review and Editing) Supervision. All authors have read and agreed to the final version of the manuscript

## Ethics Statement

The care and handling of the animals were performed in strict accordance with the recommendations of the Guide for the Care and Use of Experimental Animals of the University of Lome, Togo. The protocol was approved by the Ethics of Animal Experimentation Committee of the same university. All efforts were made to minimise discomfort to the birds (ref: 008/2021/BC‐BPA/FDS‐UL).

## Conflicts of Interest

The authors declare no conflicts of interest.

## Peer Review

The peer review history for this article is available at https://www.webofscience.com/api/gateway/wos/peer‐review/10.1002/vms3.70512.

## Data Availability

Data available in article material.
